# Are differences between groups different at different occasions?

**DOI:** 10.1007/s40037-017-0380-y

**Published:** 2017-10-25

**Authors:** Jimmie Leppink, Patricia O’Sullivan, Kal Winston

**Affiliations:** 1 Maastricht University, Maastricht, The Netherlands; 2 University of California, San Francisco, USA; 3 The Commonwealth Medical College, Scranton, PA USA

The overall purpose of the ‘Statistical Points and Pitfalls’ series is to help readers and researchers alike increase awareness of how to use statistics and why/how we fall into inappropriate choices or interpretations. We hope to help readers understand common misconceptions and give clear guidance on how to avoid common pitfalls by offering simple tips to improve your reporting of quantitative research findings. Each entry discusses a commonly encountered inappropriate practice and alternatives from a pragmatic perspective with minimal mathematics involved. We encourage readers to share comments on or suggestions for this section on Twitter, using the hashtag: #mededstats

Some studies in medical education compare groups of participants on one or more outcome variables at two or more points in time. For example, pre-test and immediate post-test performance and perhaps also a delayed post-test performance. In the majority of such studies, the interest lies in *differences between groups *over time rather than in the average score or change of a particular group. More specifically, the core research question is usually whether the difference between groups of interest changes from one occasion or time to the next. If the **difference** between **groups** is different at different **times**, we speak of a *group-by-time interaction effect*. In other words, the main research question in studies which compare groups at different occasions is usually whether there is a *group-by-time interaction effect*.

In the previous entry, we discussed that it is quite common to use statistical procedures that may provide us with no or incorrect information with regard to interaction effects [1]. In studies where groups are compared at different occasions, it is quite common to perform statistical significance tests for the difference between groups at each occasion without checking whether there is evidence for a group-by-time interaction effect or not. In this entry, we demonstrate that this practice can result in incorrect conclusions with regard to the interaction effect of interest. We conclude that when researchers are interested in a group-by-time interaction effect, they should use a statistical tool that provides an overall test for that interaction effect (e. g. repeated measures analysis) and follow up with tests for group differences at separate occasions only if that overall test provides sufficient evidence for the interaction effect of interest.

## Example study

Suppose, a team of researchers has two groups of residents practise with objective structured clinical examinations (OSCE; control group, *n* = 32) or with hypothesis-driven physical examinations [2] (HDPE; treatment group, *n* = 32) on a simulated patient in a skills lab. Right after this practice period, residents in both groups perform a physical examination on another simulated patient (i. e. immediate post-test) and return to the lab to perform a physical examination on yet another simulated patient one week later (i. e. delayed post-test). For both occasions (i. e. immediate and delayed post-test), residents are instructed to think aloud while performing the examination. Sessions are video-recorded, and two members of the skills lab who are not part of the research team and are blind to which residents have been part of which group (i. e. OSCE or HDPE) independently code students’ spoken language in terms of clinical reasoning. This yields a clinical reasoning score for each resident for each of two occasions. The researchers are interested in the question whether the two groups differ in average clinical reasoning score and hypothesize that they do differ substantially at immediate post-test but to a lesser extent at the delayed post-test (i. e. group-by-time interaction effect).

## Two scenarios

**Fig. 1:**
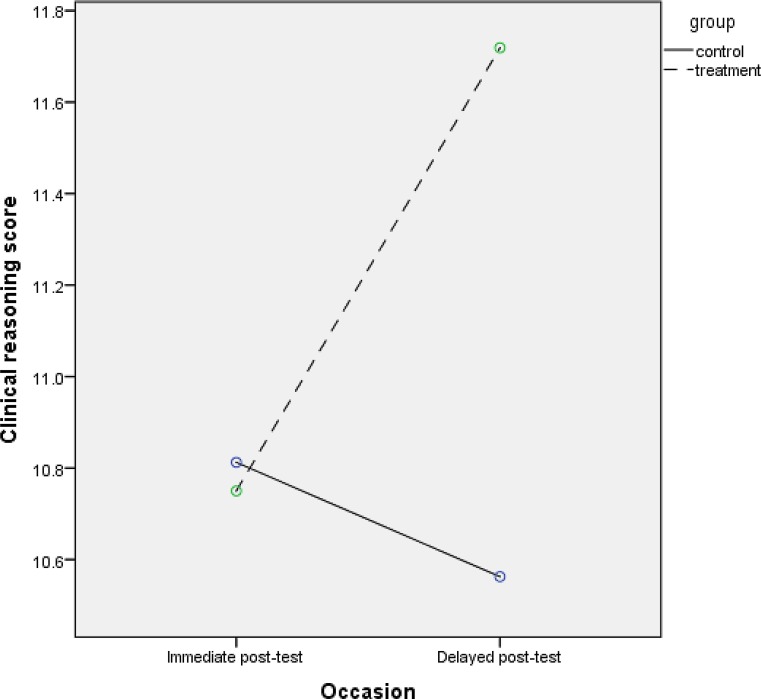
Scenario 1: group-by-time interaction effect

Figs. 1 (scenario 1) and 2 (scenario 2) illustrate two possible scenarios with regard to the outcomes of the example study. Fig. 1 depicts an example of a group-by-time interaction effect.

**Fig. 2:**
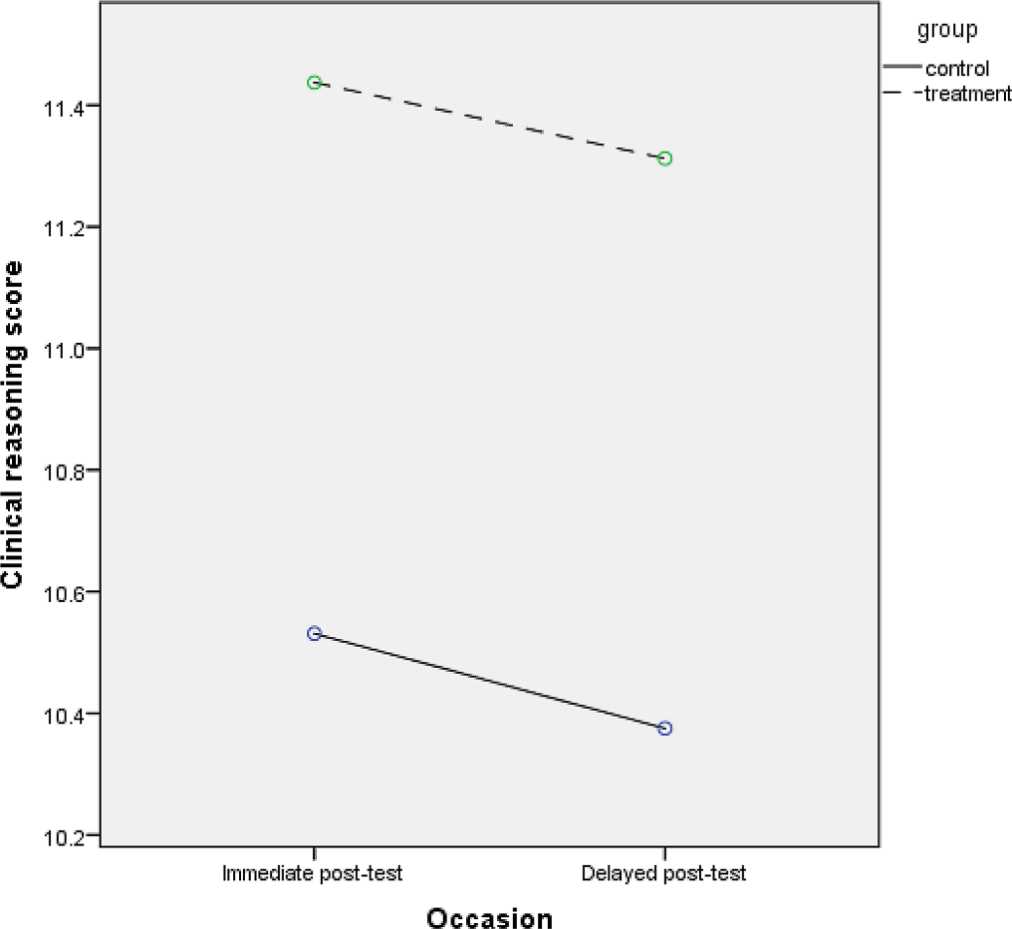
Scenario 2: main effect of group

In this scenario (1), the researchers find an average (i. e. mean) clinical reasoning score at immediate post-test of 10.81 (standard deviation, *SD* = 2.32) in the control group (OSCE) and 10.75 (*SD* = 2.00) in the treatment group (HDPE), and an average clinical reasoning score at delayed post-test of 10.56 (*SD* = 3.45) in the control group and 11.72 (*SD* = 3.27) in the treatment group. In other words, in the treatment group the average score increases with time while in the control group it does not.

Fig. 2 provides an example of a study in which there is no evidence for a group-by-time interaction effect.

In this scenario (2), the researchers find an average clinical reasoning score at immediate post-test of 10.53 (*SD* = 1.55) in the control group and 11.44 (*SD* = 1.85) in the treatment group, and an average clinical reasoning score at delayed post-test of 10.38 (*SD* = 2.45) in the control group and 11.31 (*SD* = 2.25) in the treatment group. In other words, the two groups deteriorate at about the same rate, hence the difference between groups is about the same across occasions, thus suggesting that there is no group-by-time interaction effect.

## Common incorrect approach: *t*-tests without checking for the interaction effect first

As mentioned in the introduction of this entry, quite often statistical tests for the difference between groups are performed for each occasion separately (i. e., one *t*-test for the difference between groups per occasion) without checking whether there is evidence for a group-by-time interaction effect (e. g. Fig. 1) or not (e. g. Fig. 2). Using this incorrect approach in scenario 1 yields *p* = 0.908 for the immediate post-test and *p* = 0.174 for the delayed post-test. In other words, one would have insufficient evidence to reject the null hypothesis of ‘no difference between groups’ at either occasion. Thus, one would conclude that there is no evidence for a group-by-time interaction effect, while Fig. 1 hints at such an interaction effect.

Using the incorrect approach in scenario 2 results in *p* = 0.037 for the immediate post-test and *p* = 0.116 for the delayed post-test. Hence, one would reject the null hypothesis of no difference for the immediate post-test but not for the delayed post-test. Consequently, one would conclude that there is evidence for an interaction effect, while Fig. 2 hints at no such interaction effect.

## Correct approach: check for the interaction effect first

The separate *t*-tests approach provides researchers with no or incorrect information with regard to the group-by-time interaction effect of interest. To obtain a statistical test for that interaction effect, researchers can use repeated measures analysis of variance (RM ANOVA) [3]. More specifically, RM ANOVA tests for three effects:

- *Main effect of group*: the difference between groups averaged across occasions;
- *Main effect of time*: the change from one occasion to the next averaged across groups;
- *Group-by-time interaction effect*: the extent to which the difference between groups is different at different occasions.

Since the interest typically lies in the group-by-time interaction effect rather than in one of the main effects, we recommend testing the group-by-time interaction effect first. Moreover, since the main effects in RM ANOVA are often difficult to interpret in the case of a significant group-by-time interaction effect [3], it is safe to interpret the main effects only if there is insufficient evidence for the group-by-time interaction effect.

Testing for group-by-time interaction with RM ANOVA yields *p* = 0.038 and 95% confidence interval (CI) = [0.068; 2.370] in scenario 1, and *p* = 0.950 and 95% CI = [−0.968; 1.030] in scenario 2. Hence, we reject the null hypothesis of no interaction effect in scenario 1 (95% CI does not include the null hypothesis of ‘0’ or ‘no difference’ and hence *p* < 0.05) but fail to do so in scenario 2 (95% CI includes ‘0’ and hence *p* > 0.05). In other words, while the *t*-tests approach would lead researchers to conclude a group-by-time interaction effect in scenario 2 but not in scenario 1, RM ANOVA – in line with Figs. 1 and 2 – correctly provides sufficient evidence for an interaction effect in scenario 1 but not in scenario 2. These two scenarios underline one of the core messages of our first entry in this series [4]: the importance of a numerical or graphical presentation of descriptive statistics (e. g. means and standard deviations per group per occasion) at an early stage. Moreover, these two scenarios illustrate how the *t*-tests approach can mislead researchers and audience alike with regard to group-by-time interaction.

## Scenario 1: group-by-time interaction effect

In scenario 1, RM ANOVA indicates a significant group-by-time interaction effect which is different from what the researchers expected: Fig. 1 indicates that the difference between groups at delayed post-test is larger not smaller than the difference between groups at immediate post-test. Although RM ANOVA provides an outcome with regard to whether or not a group-by-time interaction effect is statistically significant, it does not provide any information about whether the *difference* between groups *increases* or *decreases* from one occasion to the next. Moreover, this scenario illustrates that the RM ANOVA test outcome for the interaction effect is in contrast to the conclusion from the inappropriate approach of using a *t*-test for group differences per occasion initially. In other words, it is possible to find evidence for an interaction effect in RM ANOVA but no or insufficient evidence for that interaction effect in occasion-specific tests. For that reason, *t*-tests for group differences per occasion may constitute a follow-up analysis in the case of a significant interaction effect if researchers had specific a‑priori expectations with regard to the change in difference between groups from one occasion to the next, but should not be used without testing through RM ANOVA whether there is a significant interaction effect in the first place.

## Scenario 2: main effect of group

In scenario 2, RM ANOVA does not provide evidence for a group-by-time interaction effect. However, researchers who follow the incorrect approach of a separate *t*-test for group differences per occasion may erroneously conclude that there is an interaction effect, by pointing at the fact that the *t*-test yields a statistically significant difference at the immediate but not at the delayed post-test. When RM ANOVA does not provide sufficient evidence for an interaction effect, one should focus on the main effect of group in RM ANOVA. This provides a more sensible approach to testing for group differences than occasion-specific *t*-tests, because the chance of drawing incorrect conclusions with regard to group differences is smaller in RM ANOVA than in occasion-specific *t*-tests [3]. The RM ANOVA test for the main effect of group yields *p* = 0.044 and 95% CI = [0.026; 1.818]. In other words, while researchers following the incorrect approach may conclude that there is an interaction effect (*p* < 0.05 for immediate but *p* > 0.05 for delayed post-test), the correct approach provides evidence for a main effect of group (95% CI does not include ‘0’, hence *p* < 0.05) but not for the group-by-time interaction effect (95% CI includes ‘0’, hence *p* > 0.05).

## To conclude

When researchers are interested in a group-by-time interaction effect, they should use a statistical tool that provides an overall test for that interaction effect (e. g. RM ANOVA). If that overall test provides evidence for the interaction effect of interest, researchers may follow up with occasion-specific tests for group differences (e. g. *t*-tests) to study that interaction effect in more detail. If the overall test provides insufficient evidence for an interaction effect, researchers should focus on the main effect of group to test for group differences rather than occasion-specific tests for group differences.
